# Genetic assessment of a bighorn sheep population expansion in the Silver Bell Mountains, Arizona

**DOI:** 10.7717/peerj.5978

**Published:** 2018-11-30

**Authors:** John A. Erwin, Karla Vargas, Brian R. Blais, Kendell Bennett, Julia Muldoon, Sarah Findysz, Courtney Christie, James R. Heffelfinger, Melanie Culver

**Affiliations:** 1 Genetics Graduate Interdisciplinary Program, University of Arizona, Tucson, AZ, United States of America; 2 James E. Rogers, College of Law, University of Arizona, Tucson, AZ, United States of America; 3 School of Natural Resources and the Environment, University of Arizona, Tucson, AZ, United States of America; 4 Molecular and Cellular Biology Department, University of Arizona, Tucson, AZ, United States of America; 5 Arizona Game and Fish Department, Phoenix, AZ, United States of America; 6 US Geological Survey, Arizona Cooperative Fish and Wildlife Research Unit, University of Arizona, Tucson, AZ, United States of America

**Keywords:** Bighorn sheep, *Ovis canadensis*, Population growth, Microsatellites, Mitochondrial DNA, Migration, Silver Bell Mountains

## Abstract

**Background:**

The isolated population of desert bighorn sheep in the Silver Bell Mountains of southern Arizona underwent an unprecedented expansion in merely four years. We hypothesized that immigration from neighboring bighorn sheep populations could have caused the increase in numbers as detected by Arizona Game and Fish Department annual aerial counts.

**Methods:**

We applied a multilocus genetic approach using mitochondrial DNA and nuclear microsatellite markers for genetic analyses to find evidence of immigration. We sampled the Silver Bell Mountains bighorn sheep before (2003) and during (2015) the population expansion, and a small number of available samples from the Gila Mountains (southwestern Arizona) and the Morenci Mine (Rocky Mountain bighorn) in an attempt to identify the source of putative immigrants and, more importantly, to serve as comparisons for genetic diversity metrics.

**Results:**

We did not find evidence of substantial gene flow into the Silver Bell Mountains population. We did not detect any new mitochondrial haplotypes in the 2015 bighorn sheep samples. The microsatellite analyses detected only one new allele, in one individual from the 2015 population that was not detected in the 2003 samples. Overall, the genetic diversity of the Silver Bell Mountains population was lower than that seen in either the Gila population or the Morenci Mine population.

**Discussion:**

Even though the results of this study did not help elucidate the precise reason for the recent population expansion, continued monitoring and genetic sampling could provide more clarity on the genetic demographics of this population.

## Introduction

The Silver Bell Mountains of southern Arizona are home to an isolated population of Mexican desert bighorn sheep (*Ovis canadensis mexicana*) (Jansen et al., 2006). There are few nearby populations of bighorn sheep in south-central Arizona, the nearest is more than 50 km away, separated by inhospitable habitat between suitable mountain ranges, and no immigration has been recorded into the Silver Bells. The Silver Bell Mountains population is the last remnant of an endemic bighorn population complex that once included the Santa Catalina, Santa Rita, and Rincon Mountains in southeastern Arizona (Arizona Game and Fish Department, pers. comm., 2017). Though bighorn sheep were historically ubiquitous across much of the mountainous West, they suffered catastrophic declines in the late 1800s and early 1900s (Valdez & Krausman, 1999). A combination of unregulated harvest, the introduction of livestock-related diseases, and habitat alteration post-European colonization drove many bighorn sheep populations into extirpation and left fragmented remnants of formerly vibrant metapopulations in its wake (Valdez & Krausman, 1999). Though the story may sound familiar, at first glance, the population in the Silver Bells presents an interesting twist on this time-old tale: this isolated population is rapidly expanding after decades of remaining consistently small.

**Figure 1:**
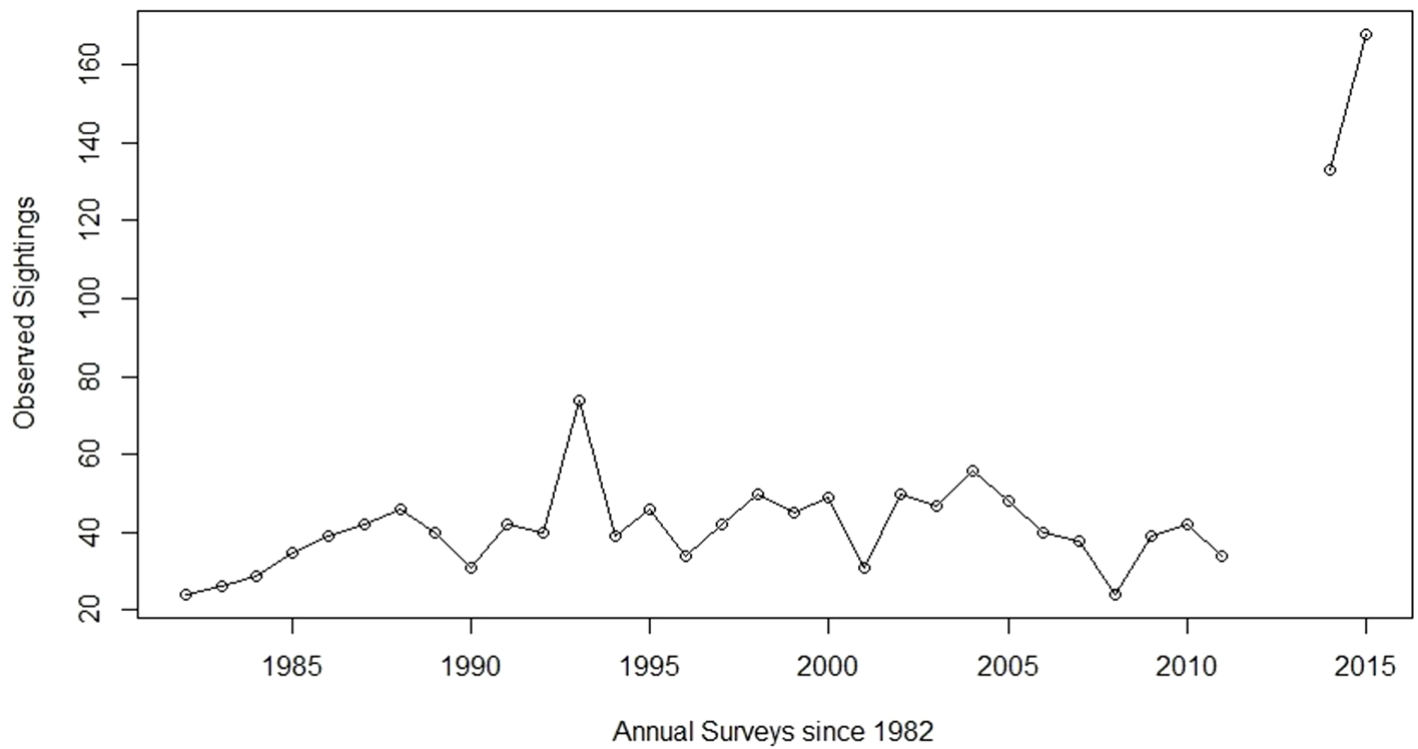
Aerial sheep surveys. Population trends from annual bighorn sheep surveys by the Arizona Game and Fish Department (AZGFD) in the Silver Bell Mountains, Arizona, USA, 1981 to 2015. No surveys were completed for 2012 and 2013. Prior to the disease outbreak, AZGFD moved to start surveying all of their sheep populations every three years, instead of annually. However, when the original outbreak occurred AZGFD resumed annual monitoring until 2012, when it seemed apparent that the disease would not reappear (A Munig, AZGFD, pers. comm., 2017).

As with all bighorn sheep populations in the state, the Arizona Game and Fish Department (AZGFD) has closely monitored the demographics and health of this population for decades. Until recently, the number of individuals in the Silver Bell bighorn sheep population had not fluctuated dramatically, at least since 1981 when monitoring began (Fig. 1). Annual aerial surveys showed a relatively stable population from 1981 to 2003, with the number of individuals observed fluctuating between 24 to 50 sheep with few exceptions (Fig. 1). In December 2003, outbreaks of infectious keratoconjunctivitis and contagious ecthyma reduced the population approximately 25% through 2008 (Jansen et al., 2007). Subsequently, the population began to slowly increase before rapidly expanding after 2011 (Fig. 1). The number of individuals observed during surveys increased from 34 sheep in 2011 to 133 and 168 in 2014 and 2015, respectively (Fig. 1). Surveys were not conducted in 2012 and 2013, due to changes in AZGFD’s statewide sheep surveying protocols. Although some previous estimates of biological potential for growth in bighorn sheep suggest a doubling of the population in four to five years (Gross, Singer & Moses, 2000; McCarty & Miller, 1998), the number of sheep observed in the Silver Bell Mountains population indicated a more dramatic increase in a four-year span.

Based on its history as an endemic population that underwent a small, albeit recent, disease-induced bottleneck in 2003, we hypothesize that a rare migration event into the population could explain the rapid expansion seen in the Silver Bell Mountains by both increasing abundance and by boosting reproductive vigor through a genetic rescue (Tallmon, Luikart & Waples, 2004; Hedrick, Adams & Vucetich, 2011; Whiteley et al., 2015).

In general, small populations are inherently more vulnerable to change in the form of environmental catastrophes, demographic changes, or stochastic environmental changes, than are large populations (Lande, 1988; Sæther et al., 2005; Willi, Van Buskirk & Hoffman, 2006). Whether isolated naturally or due to anthropogenic forces, these small populations are also more vulnerable to genetic drift, resulting in the loss of genetic diversity, fixation of alleles within populations, and reduced evolutionary potential (Frankham, 1996; Kimura, 1955; Otto & Whitlock, 1997; Wright, 1931). Additionally, inbreeding, which lowers genetic diversity by increasing homozygosity, is inevitable when the population becomes sufficiently small and isolated (Charlesworth & Charlesworth, 1999).

Small populations can often benefit in a number of ways from immigration, the introduction of individuals from other populations (Tallmon, Luikart & Waples, 2004; Whiteley et al., 2015). Immigration boosts the number of individuals, making the population more robust while also having positive genetic consequences. Specifically, genetic rescue is an increase in population fitness, as measured through demographic growth, caused by the introduction of novel alleles through immigration (Whiteley et al., 2015). Migrants may introduce novel alleles which can mask the negative effects of deleterious alleles that have built up over time (Tallmon, Luikart & Waples, 2004). The offspring of these migrants may see a boost in fitness related to higher heterozygosity, known as heterozygote advantage (Tallmon, Luikart & Waples, 2004). There is evidence that the arrival of even a single immigrant can be enough to cause the rapid spread of new alleles and exponential population growth, halting the effects of loss of heterozygosity and inbreeding depression (Vila et al., 2003). In the past, genetic rescue has improved longevity and fitness in bighorn sheep populations based on empirical evidence (Miller et al., 2012).

Despite the isolation, bighorn sheep have been known to disperse into neighboring habitats (Singer et al., 2000), making it possible that immigrant sheep could enter the Silver Bell Mountains population. Though rams and ewes have been shown to engage in natal dispersal with similar frequencies in long, linear mountain ranges (Buchalski et al., 2015), in the sky islands habitat of southern Arizona, females are believed to move less frequently between ranges, making male-biased dispersal more common (Buchalski et al., 2015; Epps et al., 2005; Festa-Bianchet, 1991). In *O. c. nelsoni*, the estimated maximum dispersal distances for rams and ewes in the Mojave Desert were ten times larger across sloped terrain (164 km and 100 km for rams and ewes respectively) than across flat terrain (16.4 km and 10 km respectively) (Epps et al., 2007; Creech et al., 2014). In other studies, dispersal over more than 60 km has been deemed unlikely (Buchalski et al., 2016). With this in mind, it is possible that the rapid growth of the Silver Bell Mountains population could be the result of immigration from other populations in Arizona.

Thus, we applied a multilocus genetic approach to determine whether immigration can at least partially explain the recent, rapid population expansion observed in the Silver Bell Mountains. If immigration was a contributing factor in expansion, we would expect to see a change in allele frequencies, and likely new alleles, in the Silver Bell Mountains population post-expansion.

We used mitochondrial DNA sequence and nuclear microsatellite markers for genetic analyses of the Silver Bell Mountains bighorn sheep before (2003) and during (2015) the population expansion. We also analyzed a small number of available blood samples from the Gila Mountains (a southwestern Arizona population) and the Morenci Mine (Rocky Mountain bighorn sheep *O. c. canadensis*) to detect the source of putative immigrants and, more importantly, to serve as comparisons for genetic diversity metrics. The Silver Bell Mountains population is of management concern for AZGFD (AZGFD, pers. comm., 2017), and we thus provide the only published population genetic analyses specifically focused on this population of concern.

## Materials and Methods

### Sampling and DNA extraction

**Figure 2:**
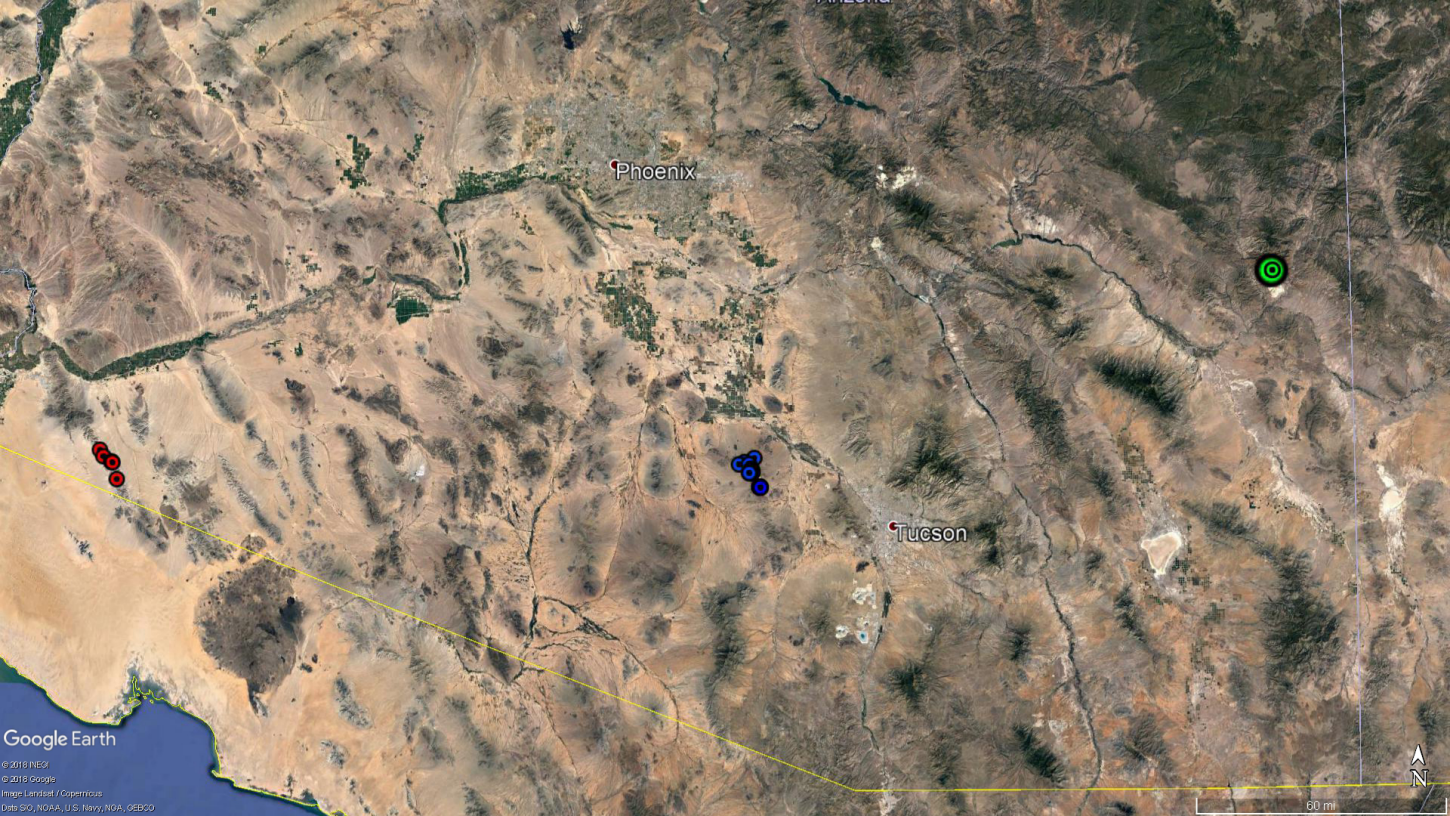
Map of bighorn sheep samples. Map of samples collected and used for microsatellite and mitochondrial DNA analyses. Red circles represent the five samples from the Gila Mountains. The blue circles represent the samples collected in the Silver Bell Mountains in 2015. Precise geographic coordinates were not available for the samples collected in the Silver Bell Mountains in 2003. Additionally, coordinates were not recorded for the samples from the Morenci Mine, so a single green circle was used to show the approximate location of that population. Map data: google Earth Pro, INEGI.

We obtained 62 bighorn sheep blood samples collected by AZGFD (Fig. 2). Of these, 52 were from the Silver Bell Mountains population (35 from the 2003 capture, when AZGFD was treating bighorn sheep for disease, and 17 from a 2015 capture, after the population had crashed and subsequently increased), five from the Gila Mountain Range, collected in 2016 from south of Highway 8 in southwestern Arizona, and 5 Rocky Mountain bighorn sheep samples collected from Morenci Mine in 2014. We used the FlexiGene DNA blood extraction kit (Qiagen, Inc., Valencia, CA, USA), and followed the manufacturer’s protocol to extract DNA from whole blood stored in EDTA tubes.

### Mitochondrial DNA

We used the primers L15712 for the forward sequence and BETH for the reverse sequence (Epps et al., 2005) to amplify a 515 base pair fragment of the mitochondrial control region. We ran 20 µL PCR reactions with the following conditions: 1×PCR Buffer (Invitrogen™, Thermo Fisher Scientific Inc. Waltham, MA, USA), 0.16 mM dNTPs, 0.05% bovine serum albumin (Sigma-Aldrich, St. Louis, MO, USA), 1.9 mM MgCl_2_, 0.4 uM of each primer, 0.8 units of Taq DNA polymerase (Invitrogen™), and 10 ng of extracted DNA. Amplification conditions were as follows: 94 °C for 7.5 min, 35 cycles of 94 °C for 60 s, 61 °C for 70 s, and 72 °C for 90 s, and a final extension at 72 °C for 7 min. We visualized PCR products by gel electrophoresis on 1.5% agarose gels stained with Ethidium Bromide. We used EXOsap-IT (USB-Affymetrix, Inc.) to purify PCR products prior to sequencing with the following PCR conditions: 37 °C for 15 min followed by 80 °C for 15 min then held at 4 °C. We sent final purified PCR products to the University of Arizona Genetics Core (UAGC) for sequencing in both the forward and reverse directions on an ABI3730 DNA Analyzer (Applied Biosystems, Thermo Fisher Scientific Inc., Waltham, MA, USA).

We used Sequencher v.5.0.1. (GeneCodes, Ann Arbor, MI) to assemble the resulting forward and reverse sequences manually. We visualized chromatograms, aligned and trimmed to a reference sequence (Genbank ID: KP688366) to resolve ambiguous reads, and exported contiguous (FASTA) sequences for analyses. We imported the sequences into MEGA v.7 (Kumar, Stecher & Tamura, 2016) to obtain a final alignment. A 75 base pair repetitive sequence was discovered to be duplicated in one haplotype, RM6. Following Buchalski et al. (2016), samples were reduced to a single copy of the repeat. We trimmed all sequences to 515 base pairs.

### Microsatellites

We used 15 microsatellite markers, which were split into two multiplexes of 8 and 7 microsatellites each (Multiplexes 1 and 2 from Buchalski et al. (2015)). For 20 uL reactions, concentrations were 1×Invitrogen reaction buffer, 2.5 mM MgCl2, 0.4 mM dNTP’s, 0.12% BSA, and 1 unit of Taq DNA Polymerase (Qiagen Inc., Valencia, CA, USA; see Buchalski et al., 2015 for primer concentrations). We used a touchdown approach for amplification conditions: 94 °C for 15 min, followed by 5 cycles of 94 °C for 30 s, annealing at 63 °C to 59 °C decreased by 1.0° per cycle for 90 s, 72 °C for 1 min, and then 30 cycles of 94 °C for 30 s, 59 °C for 90 s, 72 °C for 1 min, and a final extension at 60 °C for 30 min. We sent PCR products to the UAGC for fragment size analysis in an ABI3730 DNA analyzer (Applied Biosystems TM, Thermo Fisher Scientific Inc., Waltham, MA, USA). We used Genemarker v.2.6.0 (SoftGenetics, State College, PA, USA) to score allele sizes. Three to four samples were included as duplicates on each plate we genotyped to ensure that our allele calls were consistent.

### Data analysis

For mtDNA, we exported a final alignment from MEGA v.7 into DnaSP v.5.10.1 (Librado & Rozas, 2009). We used DnaSP to estimate the number of haplotypes, haplotype diversity, and nucleotide diversity. We used the algorithm BLAST (Altschul et al., 1990) to match mitochondrial haplotypes with haplotypes previously recorded in GenBank (Benson et al., 2018). We used PopArt to create a median neighbor joining haplotype network (Leigh & Bryant, 2015).

Three of our microsatellite markers failed to consistently amplify and as a result our final data set was reduced to 12 markers. For the nuclear microsatellites, we used GenAlEx v.6.501 (Peakall & Smouse, 2012) to determine if loci were in Hardy-Weinberg equilibrium, measure allele frequencies, and heterozygosity. Additionally, to adjust for our large disparity in sample sizes between different populations, we used HP-Rare to calculate allelic richness using rarefaction (Kalinowski, 2005). We calculated pairwise- *F*_ST_ between the four different populations using Arlequin 3.5.2.2 (Excoffier & Lischer, 2010). Calculations were based on Weir & Cockerham (1984) and statistical significance was determined using 10,000 permutations and a *p*-value of 0.05. We used GeneClass2.0 to detect first-generation migrants among each population (Piry et al., 2004). We applied the Paetkau et al. (1995) frequency-based criterion for likelihood computations, a frequency of 0.01 for missing alleles, the Paetkau et al. (2004) resampling algorithm, 10,000 resampled individuals, and a threshold significance of *p* < 0.01.

Additional analyses were run specifically in the Silver Bell populations. Probability of Identity (P_(ID)_) (Paetkau et al., 1998) and Probability of Identity assuming siblings (P_(ID)sib_) (Waits, Luikart & Taberlet, 2001) were calculated and we looked for samples with identical genotypes between time points using GenAlEx (Peakall & Smouse, 2012). Using the R package Rhh (Alho, Välimäki & Merilä, 2010), we calculated homozygosity by locus (HL) and internal relatedness (IR) for each individual (Aparicio, Ortego & Cordero, 2006). Welch’s *t*-test was used to determine whether or not differences between HL and IR for the two temporal samples were significantly different. Finally, we used ML-Relate to calculate the pairwise relatedness (r) between each individual in each temporal sample (Kalinowski, Wagner & Taper, 2006).

We used the Bayesian clustering procedure implemented in STRUCTURE v2.3.2.1 (Hubisz et al., 2009) to genetically assign individuals to clusters, assuming admixture and correlated allele frequencies between populations. We considered the number of populations (*K*) from 1 to 10. We ran STRUCTURE with 1,000,000 Markov chain Monte Carlo iterations and 20 independent simulations per *K* with the first 200,000 iterations eliminated as burn-in. We used the Δ*K* statistic of Evanno, Regnaut & Goudet (2005) implemented in the CLUMPAK server (clumpak.tau.ac.il; Kopelman et al., 2015) to determine the most appropriate number of genetic clusters.

**Figure 3:**
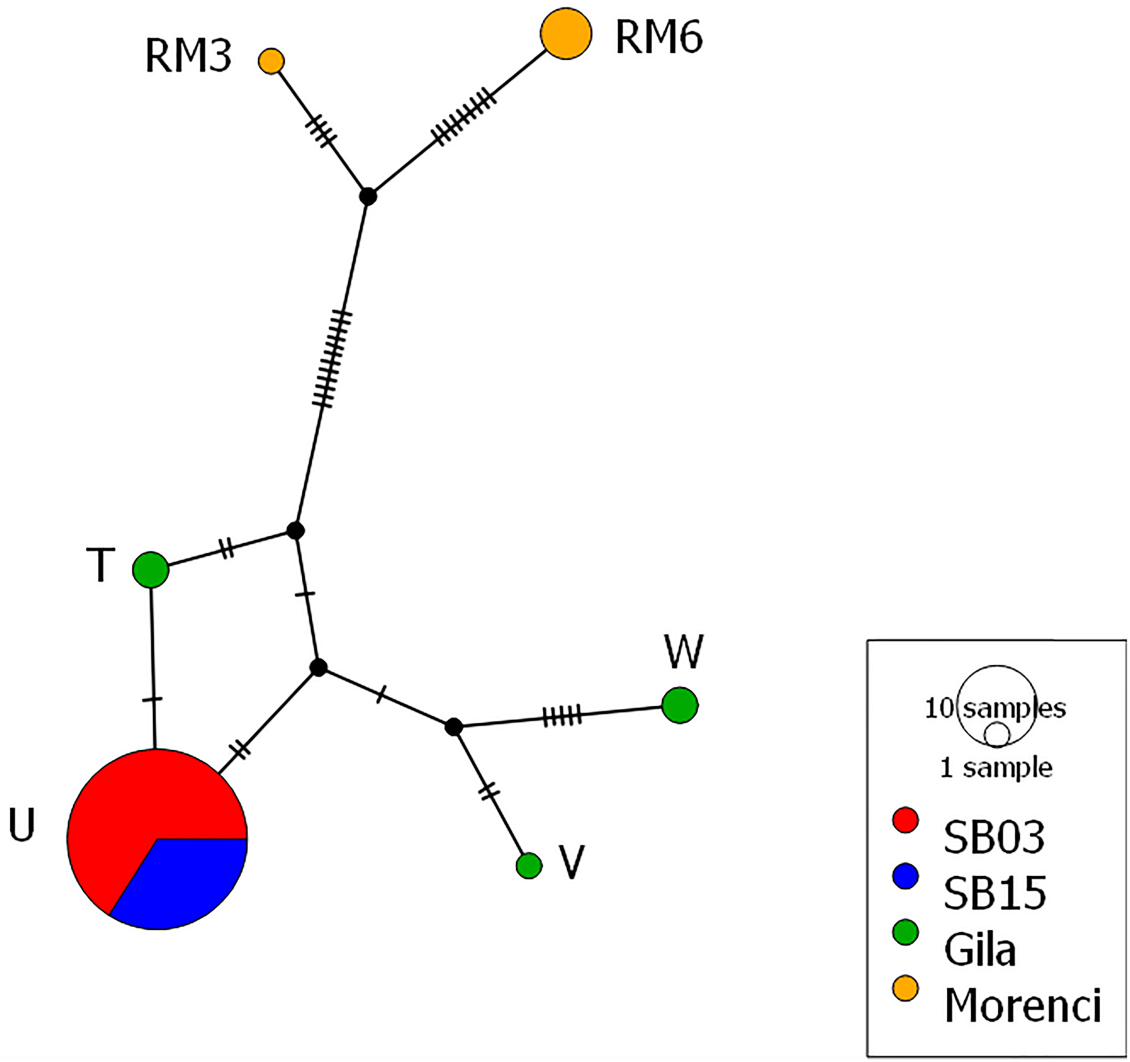
Mitochondrial haplotype network. Each circle represents one of the six different haplotypes found and each haplotype is labeled with the haplotype name previously published and uploaded to GenBank. Each hash mark along each branch represents one base change and the size of the circle corresponds to the number of individuals. Both the 2003 and 2015 time points from the Silver Bell Mountains (colored red and blue respectively) share the same haplotype. There are three haplotypes from the Gila population (colored green) and two haplotypes from the Morenci Mine population (colored orange). The Silver Bell Mountains haplotype appears much more similar to those from the Gila population than the Morenci Mine population. Each haplotype matched a previously deposited sequence in GenBank, as follows: Haplotype U –AY904014, Haplotype W –AY904016, Haplotype T –AY904013, Haplotype V –AY904015, Haplotype RM6 –KU363685, and Haplotype RM3 –KU363682. The haplotype network was created in PopArt (Leigh & Bryant, 2015).

**Table 1:** Population genetic statistics. All statistics were generated in GenAlEx unless otherwise noted. *N* is the number of samples. *A*_*r*_ is allelic richness as calculated in HP-Rare. *A*_*ne*_ is the effective number of alleles. *A*_*p*_ is the average number of private alleles. *H*_*o*_ is the observed heterozygosity and *uH*_*e*_ is the unbiased expected heterozygosity. *F*_*IS*_ is the inbreeding coefficient. *HL* is the average homozygosity by locus and *IR* is the average internal relatedness, as both calculated with Rhh. For the mitochondrial DNA, *H* is the number of haplotypes, *H*_*d*_ is the haplotype diversity, and Π is nucleotide diversity, calculated in PopArt. Populations: SB2003 are Silver Bell Mountains samples from 2003. SB2015 are Silver Bell Mountains samples from 2015. Gila are samples from the Gila Mountains. Morenci are samples from the Morenci Mine population.

Population	Microsatellites									Mitochondria		
	*N*	*A*_R_	*A*_ne_	*A*_p_	*H*_o_	*uH*_e_	*F*_IS_	*HL*	*IR*	*H*	*H*_d_	Π
SB2003	35	1.54	1.36	0.17	0.18	0.19	0.014	0.56	0.056	1	0	0
SB2015	17	1.57	1.40	0	0.21	0.21	0.001	0.54	−0.005	1	0	0
Gila	5	2.33	1.86	0.67	0.45	0.43	−0.143	–	–	3	0.8	0.012
Morenci	5	2.92	2.17	1.83	0.52	0.52	−0.052	–	–	2	0.4	0.010

## Results

From the mitochondrial DNA, we observed six haplotypes among the collected samples, all of which were matched to known haplotypes from GenBank (Fig. 3). We found three haplotypes within the Gila population, and two haplotypes from the Morenci Mine population. The remaining haplotype was shared in all samples from the Silver Bell Mountains, from both 2003 and 2015. There were fewer base pair differences between the Silver Bell Mountains population and the Gila population, both of which are *O. c. mexicana*, than the Morenci Mine population, which is *O. c. canadensis* (Fig. 3). On average, haplotype diversity was 0.299, and the nucleotide diversity was 0.007 (Table 1).

At the microsatellite loci, the sheep from the Silver Bell Mountains had lower genetic diversity (Table 1). Furthermore, sheep from the Silver Bell Mountains had lower allelic richness (1.54 in 2003 and 1.57 in 2015) than those from the Gila Mountains (2.33) and Rocky Mountain bighorn sheep from Morenci Mine (2.92). The Silver Bell Mountains population showed a much lower unbiased expected heterozygosity (0.19 for 2003 and 0.21 for 2015) than the Gila and Morenci Mine populations (0.43 and 0.52 respectively). *F*_IS_ values from all populations were near zero, with −0.052 for Morenci Mine, −0.143 for Gila, 0.001 for the Silver Bell Mountains in 2015, and 0.014 for the Silver Bell Mountains in 2003. Additionally, all pairwise *F*_ST_ estimates were significant, except for the comparison between the 2003 and 2015 Silver Bell Mountains sheep (Table 2). We did not detect any statistically significant first-generation migrants with GeneClass2.0.

**Table 2:** Pairwise *F*_*ST*_ values for each population. This table shows the pairwise *F*_*ST*_ values between each of the populations as calculated in Arlequin 3.5.2.2 . Significance was tested with 10,000 permutations and *F*_*ST*_ values were calculated using Weir & Cockerham (1984).

SB2003	SB2015	Gila	Morenci	
0.000				SB2003
0.02	0.000			SB2015
0.244^*^	0.171^*^	0.000		Gila
0.644^*^	0.591^*^	0.377^*^	0.000	Morenci

**Notes.**

* Significant values (*p* < 0.05) are denoted with an asterisk.

**Figure 4:**
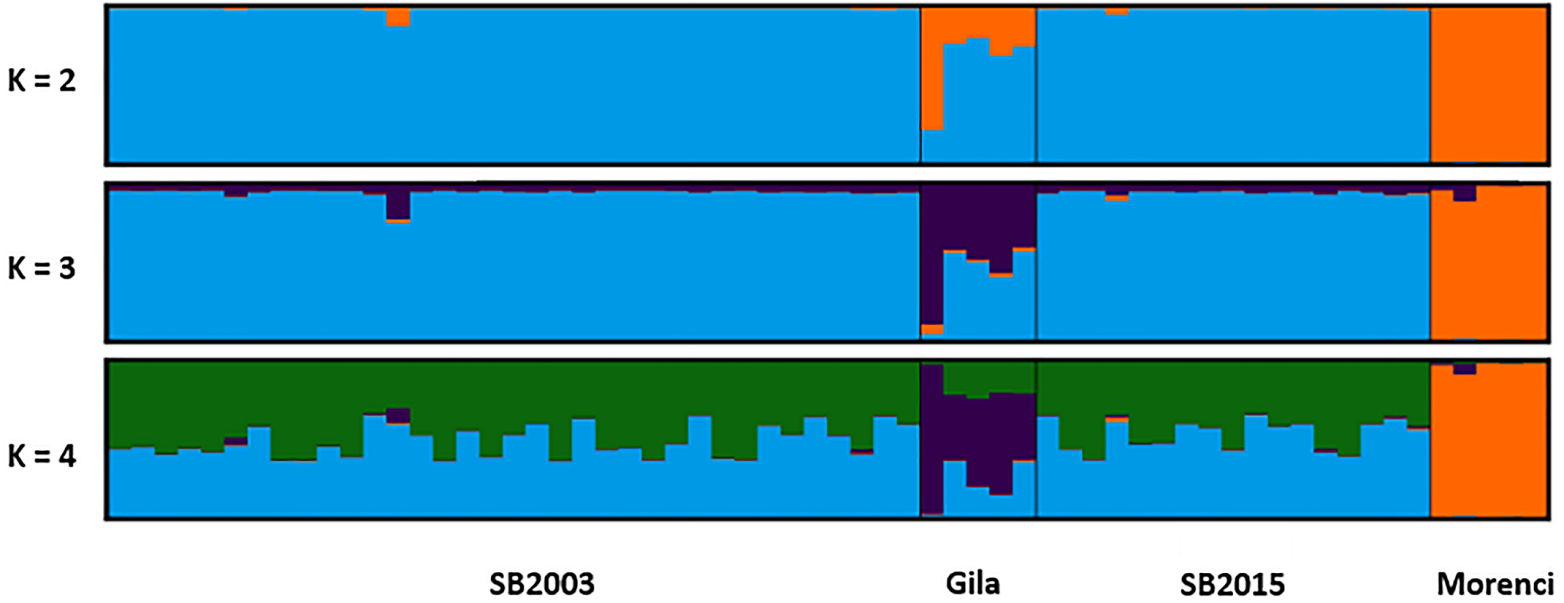
STRUCTURE plot. The bar plots display the probability of assignment into *K* = 2 − 4 population clusters, respectively, with each column representing each individual sample. Evanno’s ΔK method was used to determine the most probable number of clusters (Fig. S1). Though *K* = 2 and *K* = 4 are best supported, *K* = 3 is likewise included as the most biologically relevant *K*, based on our sampling scheme. Critically, we detected no admixture in the 2015 Silver Bell Mountains population (SB2015) when compared to the 2003 Silver Bell Mountains population (SB2003).

The two temporally separate sample sets from the Silver Bell Mountains population were similar genotypically, but not identical. The 2003 sheep had two alleles, both at locus MAF214, which did not appear in the 2015 population. Conversely, we did detect one allele in an individual in the 2015 population that was not detected in any of the 2003 sheep sampled. Average pairwise genetic relatedness (r) between individuals was comparable for each temporal sample (0.198 for 2003 and 0.176 for 2015). Additionally, average HL for each temporal sample was measured at 0.56 in 2003 and 0.54 in 2015; the Welch’s *t*-test suggested that this difference was not significant (*p* = 0.815). Likewise, average IR for each temporal sample equaled 0.056 in 2003 and then −0.005 in 2015, yet again this was a non-significant difference (*p* = 0.624). The STRUCTURE plot failed to detect a significant shift in allele frequencies between the 2003 and 2015 Silver Bell Mountains bighorn sheep population as well (Fig. 4). Finally, P_(ID)_ was calculated as 7.6 ×10^−3^ and 5.1 ×10^−3^, for 2003 and 2015. P_(ID)sib_ was calculated as 9.2 ×10^−2^ and 7.2 ×10^−2^ respectively. Six samples in 2003 had a matching genotype to a sample in 2015, and there were 3 pairs of samples in 2003 that had the same genotype at every locus. It is possible that some sheep were resampled in 2015, but our detection thresholds are too high to rule out the possibility that these were different individuals, especially since we found some identical genotypes in just the 2003 samples.

## Discussion

Our original hypothesis to explain the population expansion seen in the Silver Bell Mountains was that immigration into the population resulted in the observed expansion through both numerically increasing the number of sheep and affecting a genetic rescue. However, we are unable to support this hypothesis. Based on the genetic data, we failed to support findings of substantial gene flow into the population. We found no new mitochondrial haplotypes in the 2015 sheep from the Silver Bell Mountains and only a single new microsatellite allele, found in one individual. While the lack of novel mitochondrial diversity is less surprising, based on the general pattern of male-biased dispersal in desert bighorn sheep, the microsatellites would pick up novel alleles introduced by male immigrants. It is likely that the one new microsatellite allele was simply not measured in the 2003 population due to its low frequency and the sample sizes involved. Interestingly, this rare allele was prevalent in the Rocky Mountain bighorn sheep from the Morenci Mine site; yet no other evidence from the mitochondrial or nuclear genetics supports immigration from the Morenci Mine population. GeneClass2.0 did not highlight a single sample in the 2015 population as a likely first-generation migrant, though this analysis is limited by only having the two potential source populations for migrants.

The Silver Bell Mountains population of bighorn sheep did show some genetic signals of being small and isolated. Genetic diversity was much lower in the Silver Bell Mountains than seen in either the Gila population or the Morenci Mine population; there was more diversity present in just those five samples than in the entire Silver Bell Mountains. This finding, in spite of our low sample size (only five samples in each of those two populations) is worrying for the Silver Bell Mountain sheep population. However, concerns about the Silver Bell Mountains population being potentially inbred do not seem warranted based on our data. *F*_IS_, HL, IR and r all provide some support that this population, despite its small size for many generations seemingly without immigration, has managed to mostly avoid inbreeding. For example, other isolated, bottlenecked populations of desert bighorn have much higher estimated inbreeding coefficients: Red Rock, AZ at 0.390 and Tiburon Island, MX at 0.288 (Hedrick, 2014) compared to the Silver Bell Mountains at 0.001. While we did not detect genetic signals of inbreeding, immigration would still likely be beneficial to the genetic health of the Silver Bell Mountains population, especially in the face of its low genetic diversity and continued isolation.

While we did not detect gene flow into the Silver Bell Mountains, we cannot definitively reject the possibility that immigration has occurred, especially in recent years. The lack of new mitochondrial haplotypes only rules out immigration from females with a new haplotype, meaning males, or females with this same haplotype, could have entered the population. The haplotype observed in the Silver Bell Mountains is not unique and it has been observed elsewhere in Arizona and California (Buchalski et al., 2016; Epps et al., 2005). The lack of novel microsatellite alleles, on the other hand, is strong evidence for the lack of gene flow into this population. However, if gene flow into the Silver Bell Mountains population occurred from a population with a very similar genetic makeup it might be undetectable with our methods but may be differentiated enough to provide a slight increase in diversity and improved fitness. Additionally, functional variation could have been introduced into the Silver Bell population and played a part in the recovery. We would not have been able to detect functional variation in the nuclear genome, as we only used neutral microsatellites. An increase in MHC diversity could have played a part in the expansion, as this population had a recent history of disease-induced bottlenecks.

Our inability to detect novel alleles or changes in allele frequencies in the Silver Bell Mountains population before and after the population boom could alternatively be a function of timing. Introgression into the population, especially if from a closely related population, would see the novel alleles slowly filter through the populations (Johnson et al., 2010). By sampling in 2015, the novel variation could simply be too low in frequency to have been detected with our sample size and number of markers. For example, when Florida panthers (*Puma concolor*) were rescued by an introduction of pumas from Texas, it took a minimum of 3 years, about one full generation (Logan & Sweanor, 2001), before Texas alleles became detectable in the Florida population (Johnson et al., 2010). With that being said, generation time for bighorn sheep is usually considered to be about 6 years (Coltman et al., 2003;  Johnson et al., 2011). Generally, once a ewe reaches the age of two or three, they birth one or, rarely, two lambs per year (Monson & Sumner, 1980). Rams, on the other hand, are generally not successful breeders until the age of four (Monson & Sumner, 1980). If we assume that two generations have occurred during that time frame, it is possible that this is too few generations post-expansion to see changes in allele frequencies at only 12 microsatellite markers. However, Epps, Crowhurst & Nickerson (2018) detected the influx of migrants into desert bighorn sheep populations over just two generations (2003–2015) using a similar number (15) of microsatellite markers. They found significant changes in genetic structure due to immigration and were able to determine which sheep were immigrants and the populations from which they originated (Epps, Crowhurst & Nickerson, 2018). In order to detect potential evidence of immigration that we could have missed with our study design, a future study could employ some combination of more microsatellites markers, larger sample size post-expansion, and sampling generations into the future. A study of adaptive variation, whether that be through genome wide SNPs or specifically the MHC, could elucidate potential differences in functional genes that have fitness consequences.

Alternatively, if no immigrants entered into the population, then the Silver Bell Mountains bighorn sheep population could have expanded due to a change in some environmental variables. Precipitation (Bender & Weisenberger, 2005), escape cover (Dunn, 1996), predation (Hayes et al., 2000), forage quantity and quality (DeYoung et al., 2000), and disease (Gross, Singer & Moses, 2000) all have been shown to have effects on growth of bighorn sheep populations, and thus some potentially undetected change to these environmental conditions could have, in theory, driven the growth instead. At full biotic capacity, bighorn sheep are believed to be capable of doubling their population size every 4–5 years (McCarty & Miller, 1998), yet based on standardized, repeated aerial helicopter surveys, the number of sheep observed in the Silver Bell Mountains nearly quadrupled in that same time frame. The observed population growth is exceptional, even accounting for wide margins of error due to survey methodology.

## Conclusions

Population genetic comparison of two time points and two potential source herds using 12 microsatellite loci and a mitochondrial marker did not support immigration as an explanation for population expansion. We hypothesized that we would find an empirical example of unassisted genetic rescue in the wild, yet we failed to find evidence that the molecular benefits of immigration attributed to this dramatic population growth and expansion. Though we did not explain this phenomenon, we do offer the first published genetic characterization of this population of concern. Continued monitoring (abundance, habitat and environmental variables, health characteristics such as disease, etc.) is more important than ever in the face of this intriguing population trend, especially as we demonstrated that the Silver Bell Mountains population has reduced genetic diversity. Determining whether the population is continuing to grow, is at carrying capacity, or if it will disperse to adjacent mountain ranges could be important for determining why the population growth occurred in the first place. Based on our mitochondrial and nuclear genetic analyses, this population expansion does not appear to be directly linked to a genetic or demographic effect of immigration, though increased sampling efforts with more individuals, potential source populations, and non-neutral genetic markers over a longer time period could provide a clearer picture. An environmentally-linked driver such as changes in habitat, predation, or something else entirely has likely resulted in this population expansion, though we cannot emphatically reject our original hypothesis of genetic rescue.

##  Supplemental Information

10.7717/peerj.5978/supp-1 Figure S1 Delta *K* graph for STRUCTURE This is the Delta *K* graph used to select which *K* values best fit the model. We used the *K* statistic of Evanno, Regnaut & Goudet (2005) implemented in the CLUMPAK server (clumpak.tau.ac.il; Kopelman et al., 2015) to determine the most appropriate number of genetic clusters.

10.7717/peerj.5978/supp-2 Supplemental Information 1 Raw microsatellite calls

## Additional Information and Declarations

### Data Availability

The following information was supplied regarding data availability: The raw data are provided in a Supplemental File.

## References

[ref-1] Alho JS, Välimäki K, Merilä J (2010). Rhh: an R extension for estimating multilocus heterozygosity and heterozygosity-herterozygosity correlation. Molecular Ecology Resources.

[ref-2] Altschul SF, Gish W, Miller W, Myers EW, Lipman DJ (1990). Basic local alignment search tool. Journal of Molecular Biology.

[ref-3] Aparicio JM, Ortego J, Cordero PJ (2006). What should we weigh to estimate heterozygosity, alleles or loci?. Molecular Ecology.

[ref-4] Bender LC, Weisenberger ME (2005). Precipitation, density, and population dynamics of desert bighorn sheep on San Andres National Wildlife Range, New Mexico. Wildlife Society Bulletin.

[ref-5] Benson DA, Cavanaugh M, Clark K, Karsch-Mizrachi I, Ostell J, Pruitt KD, Sayers EW (2018). GenBank. Nucleic Acids Research.

[ref-6] Buchalski MR, Navarro AY, Boyce WM, Vickers TW, Tobler MW, Nordstrom LA, García JA, Gille DA, Penedo MCT, Ryder OA, Ernest HB (2015). Genetic population structure of Peninsular bighorn sheep (*Ovis canadensis nelsoni*) indicates substantial gene flow across US-Mexico border. Biological Conservation.

[ref-7] Buchalski MR, Sacks BN, Gille DA, Penedo MCT, Ernest HB, Morrison SA, Boyce WM (2016). Phylogeographic and population genetic structure of bighorn sheep (*Ovis canadensis*) in North American deserts. Journal of Mammalogy.

[ref-8] Charlesworth B, Charlesworth D (1999). The genetic basis of inbreeding depression. Genetical Research.

[ref-9] Coltman DW, O’Donoghue P, Jorgenson JT, Hogg JT, Strobeck C, Festa-Bianchet M (2003). Undesirable evolutionary consequences of trophy hunting. Nature.

[ref-10] Creech TG, Epps CW, Monello RJ, Wehausen JD (2014). Using network theory to prioritize management in a desert bighorn sheep metapopulation. Landscape Ecology.

[ref-11] DeYoung RW, Hellgren EC, Fullbright TE, Robbins WF, Humphreys ID (2000). Modeling nutritional carrying capacity for translocated desert bighorn sheep in western Texas. Restoration Ecology.

[ref-12] Dunn WC (1996). Evaluating bighorn habitat: a landscape approach. Technical Note 395.

[ref-13] Epps CW, Crowhurst RS, Nickerson BS (2018). Assessing changes in functional connectivity in a desert bighorn sheep metapopulation after two generations. Molecular Ecology.

[ref-14] Epps CW, Palsboll PJ, Wehausen JD, Roderick GK, Ramey RR, McCullough DR (2005). Highways block gene flow and cause a rapid decline in genetic diversity of desert bighorn sheep. Ecology Letters.

[ref-15] Epps CW, Wehausen JD, Bleich VC, Torres SG, Brashares JS (2007). Optimizing dispersal and corridor models using landscape genetics. Journal of Applied Ecology.

[ref-16] Evanno G, Regnaut S, Goudet J (2005). Detecting the number of clusters of individuals using the software STRUCTURE: a simulation study. Molecular Ecology.

[ref-17] Excoffier L, Lischer HEL (2010). Arlequin suite ver 3.5: a new series of programs to perform population genetics analyses under Linux and Windows. Molecular Ecology Resources.

[ref-18] Festa-Bianchet M (1991). The social system of bighorn sheep: grouping patterns, kinship and female dominance rank. Animal Behavior.

[ref-19] Frankham R (1996). Relationship of genetic variation to population size in wildlife. Conservation Biology.

[ref-20] Gross JE, Singer FJ, Moses ME (2000). Effects of disease, dispersal, and area on bighorn sheep restoration. Restoration Ecology.

[ref-21] Hayes CL, Rubin ES, Jorgensen MC, Botta RA, Boyce WM (2000). Mountain lion predation of bighorn sheep in the peninsular ranges, California. Journal of Wildlife Management.

[ref-22] Hedrick PW (2014). Conservation genetics and the persistence and translocation of small populations: bighorn sheep populations as examples. Animal Conservation.

[ref-23] Hedrick PW, Adams JR, Vucetich JA (2011). Reevaluating and broadening the definition of genetic rescue. Conservation Biology.

[ref-24] Hubisz MJ, Falush D, Stephens M, Pritchard JK (2009). Inferring weak population structure with the assistance of sample group information. Molecular Ecology Resources.

[ref-25] Jansen BD, Heffelfinger JR, Noon TH, Krausman PR, DeVos Jr JC (2006). Infectious keratoconjunctivitis in bighorn sheep, Silver Bell Mountains, Arizona, USA. Journal of Wildlife Diseases.

[ref-26] Jansen BD, Krausman PR, Heffelfinger JR, DeVos Jr JC (2007). Influence of mining on behavior of bighorn sheep. Southwestern Naturalist.

[ref-27] Johnson HE, Mills LS, Wehausen JD, Stephenson TR, Luikart G (2011). Translating effects of inbreeding depression on component vital rates to overall population growth in endangered bighorn sheep. Conservation Biology.

[ref-28] Johnson WE, Onorato DP, Roelke ME, Land ED, Cunningham M, Belden RC, McBride R, Jansen D, Lotz M, Shindle D, Wildt DE, Penfold LM, Hostetler JA, Oli MK, O’Brien SJ (2010). Genetic restoration of the Florida panther. Science.

[ref-29] Kalinowski ST (2005). HP-RARE 1.0: a computer program for performing rarefaction on measures of allelic richness. Molecular Ecology Notes.

[ref-30] Kalinowski ST, Wagner AP, Taper ML (2006). ML-Relate: a computer program for maximum likelihood estimation of relatedness and relationship. Molecular Ecology Notes.

[ref-31] Kimura M (1955). Solution of a process of random genetic drift with a continuous model. Proceedings of the National Academy of Sciences of the United States of America.

[ref-32] Kopelman NM, Mayzel J, Jakobsson M, Rosenberg NA, Mayrose I (2015). CLUMPAK: a program for identifying clustering modes and packaging population structure inferences across K. Molecular Ecology Resources.

[ref-33] Kumar S, Stecher G, Tamura K (2016). MEGA7: molecular evolutionary genetics analysis version 7.0 for bigger datasets. Molecular Biology and Evolution.

[ref-34] Lande R (1988). Genetics and demography in biological conservation. Science.

[ref-35] Leigh JW, Bryant D (2015). PopART: full-feature software for haplotype network construction. Methods in Ecology and Evolution.

[ref-36] Librado P, Rozas J (2009). DnaSP v5: a software for comprehensive analysis of DNA polymorphism data. Bioinformatics.

[ref-37] Logan KA, Sweanor LL (2001). Desert puma: evolutionary ecology and conservation of an enduring carnivore.

[ref-38] McCarty CW, Miller MW (1998). Modeling the population dynamics of bighorn sheep: a synthesis of literature. Colorado Division of Wildlife Special Report.

[ref-39] Miller JM, Poissant J, Hogg JT, Coltman DW (2012). Genomic consequences of genetic rescue in an insular population of bighorn sheep (*Ovis canadensis*). Molecular Ecology.

[ref-40] Monson G, Sumner L (1980). The Desert Bighorn: its life history, ecology & management.

[ref-41] Otto SP, Whitlock MC (1997). The probability of fixation in populations of changing size. Genetics.

[ref-42] Paetkau D, Calvert W, Sterling I, Strobeck C (1995). Microsatellite analysis of population structure in Canadian polar bears. Molecular Ecology.

[ref-43] Paetkau D, Slade R, Burden M, Estoup A (2004). Direct, real-time estimation of migration rate using assignment methods: a simulation-based exploration of accuracy and power. Molecular Ecology.

[ref-44] Paetkau D, Waits LP, Clarkson PL, Craighead L, Vyse E, Ward R, Strobeck C (1998). Variation in genetic diversity across the range of North American brown bears. Conservation Biology.

[ref-45] Peakall R, Smouse PE (2012). GenAlEx 6.5: genetic analysis in Excel. Population genetic software for teaching and research-an update. Bioinformatics.

[ref-46] Piry S, Alapetite A, Cornuet JM, Paetkau D, Baudouin L, Estoup A (2004). GENECLASS2: a software for genetic assignment and first-generation migrant detection. Journal of Heredity.

[ref-47] Sæther BE, Engen S, Møller AP, Visser ME, Matthysen E, Wolfgang F, Lambrechts MM, Becker PH, Brommer JE, Dickinson J, Du Feu C, Gehlbach FR, Merilä J, Rendell W, Robertson RJ, Thomson D, Török J (2005). Time to extinction of bird populations. Ecology.

[ref-48] Singer FJ, Moses ME, Bellew S, Sloan W (2000). Correlates to colonizations of new patches by translocated populations of bighorn sheep. Restoration Ecology.

[ref-49] Tallmon DA, Luikart G, Waples RS (2004). The alluring simplicity and complex reality of genetic rescue. Trends in Ecology & Evolution.

[ref-50] Valdez R, Krausman PR, Valdez R, Krausman PR (1999). Description, distribution, and abundance of mountain sheep in North America. Mountain sheep of North America.

[ref-51] Vila C, Sundqvist AK, Flagstad O, Seddon J, Björnerfeldt S, Kojola I, Casulli A, Sand H, Wabakken P, Ellegren H (2003). Rescue of a severely bottlenecked wolf (*Canis lupus*) population by a single immigrant. Proceedings of the Royal Society B-Biological Sciences.

[ref-52] Waits LP, Luikart G, Taberlet P (2001). Estimating the probability of identity among genotypes in natural populations: cautions and guidelines. Molecular Ecology.

[ref-53] Weir BS, Cockerham C (1984). Estimating F-statistics for the analysis of population structure. Evolution.

[ref-54] Whiteley AR, Fitzpatrick SW, Funk WC, Tallmon DA (2015). Genetic rescue to the rescue. Trends in Ecology & Evolution.

[ref-55] Willi Y, Van Buskirk J, Hoffman AA (2006). Limits to the adaptive potential of small populations. Annual Review of Ecology, Evolution, and Systematics.

[ref-56] Wright S (1931). Evolution in Mendelian populations. Genetics.

