# Supplementary figures and images for: Genetic assessment of a bighorn sheep population expansion in the Silver Bell Mountains, Arizona
Source: PeerJ. 2018 Nov 30;6:e5978. doi: 10.7717/peerj.5978 (PMC6276589; doi:10.7717/peerj.5978)

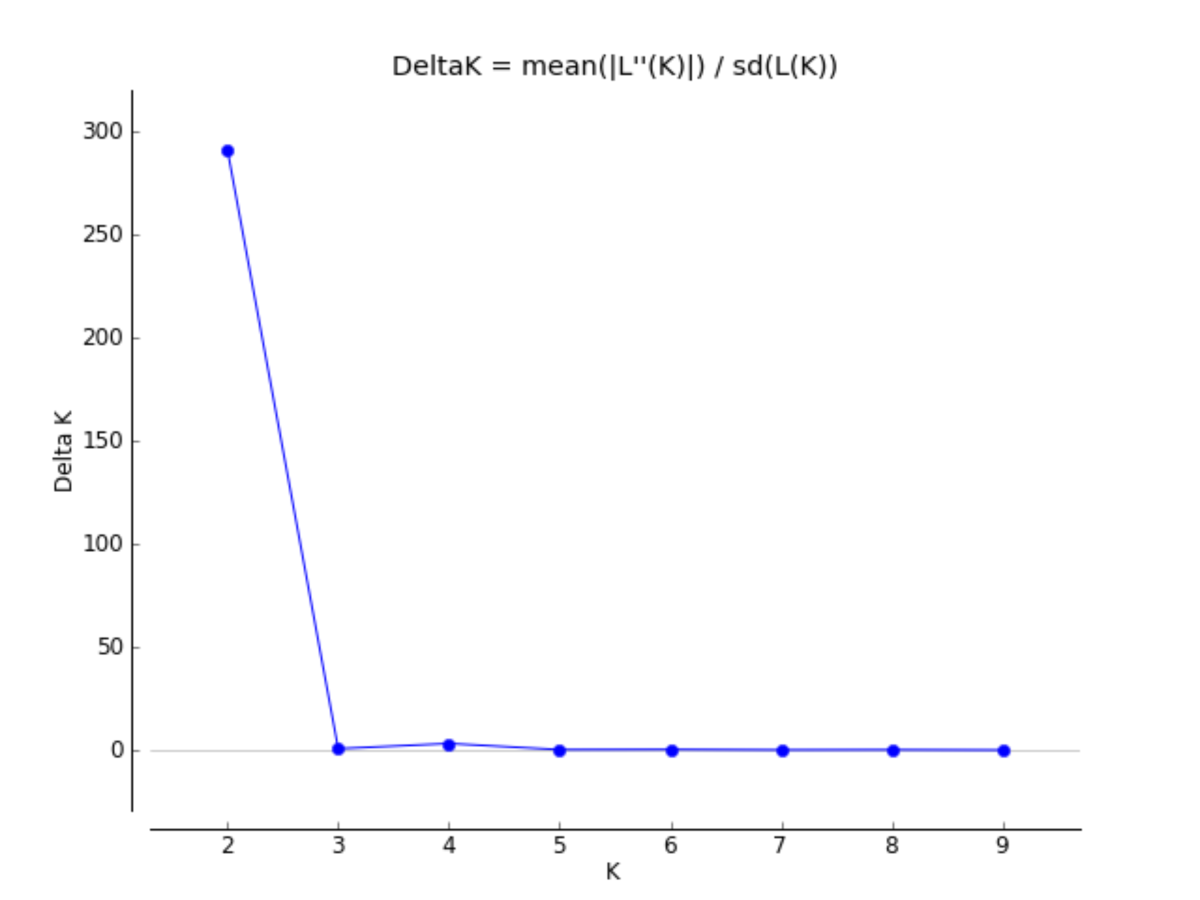

Supplement: Figure S1 — This is the Delta K graph used to select which K values best fit the model. We used the K statistic of Evanno, Regnaut & Goudet (2005) implemented in the CLUMPAK server (clumpak.tau.ac.il; Kopelman et al., 2015) to determine the most appropriate number of genetic clusters. [file peerj-06-5978-s001.png]
